# Age- and sex-specific associations of underweight and excess adiposity with incident CKD in type 2 diabetes: nationwide Korean cohort study

**DOI:** 10.3389/fendo.2026.1928884

**Published:** 2026-09-17

**Authors:** Seung Min Chung, Bongseong Kim, Mi Kyung Kim, Kyungdo Han, Nan Hee Kim

**Affiliations:** 1 Division of Endocrinology and Metabolism, Department of Internal Medicine, Yeungnam University College of Medicine, Daegu, Republic of Korea; 2 Department of Statistics and Actuarial Science, Soongsil University, Seoul, Republic of Korea; 3 Division of Endocrinology & Metabolism, Department of Internal Medicine, Keimyung University Dongsan Medical Center, Daegu, Republic of Korea; 4 Division of Endocrinology and Metabolism, Department of Internal Medicine, Korea University Ansan Hospital, Ansan, Republic of Korea

**Keywords:** chronic kidney disease, clinical obesity, diabetic nephro pathy, excess adiposity, type 2 diabetes mellitus

## Abstract

**Background:**

Obesity is a well-established risk factor for diabetic chronic kidney disease (CKD), but the relationship between body mass index (BMI) and CKD risk remains inconsistent, with paradoxical findings in underweight individuals. These discrepancies may reflect age- and sex-related heterogeneity. Following the Lancet Commission recommendation to integrate adiposity measures, including BMI and waist circumference (WC), we assessed the association between adiposity and incident CKD by age and sex in Korean adults with type 2 diabetes mellitus (T2DM).

**Methods:**

Using data from 1,824,317 adults aged ≥40 years with T2DM in the Korean National Health Insurance Service database (2015–2016), excluding those with proteinuria or impaired kidney function, participants were classified by combined BMI and WC as underweight (1.0%), no obesity (59.3%), and excess adiposity (39.6%). Incident CKD was defined by age-adjusted estimated glomerular filtration rate thresholds: new-onset eGFR <60 mL/min/1.73 m^2^ (age 40-64) and eGFR <45 mL/min/1.73 m^2^ (age ≥65).

**Results:**

During a 4-year follow-up, CKD incidence was 2.8%, 2.3%, and 2.9% across underweight, no obesity, and excess adiposity groups, respectively. Underweight was associated with a substantially greater risk of incident CKD than excess adiposity (OR 1.41, 95% CI 1.29–1.54 vs. OR 1.03, 95% CI 1.01–1.05). Age- and sex-stratified analyses revealed that underweight was associated with increased CKD risk across age and sex groups, particularly in middle-aged men, whereas excess adiposity was associated with increased risk in women but not in men.

**Conclusions:**

In Korean adults aged ≥40 years with T2DM, underweight consistently predicted higher CKD risk, particularly in middle-aged men, whereas the association of excess adiposity with CKD differed by age and sex. These findings may help explain previously inconsistent associations between obesity and CKD risk.

## Background

The prevalence of obesity among individuals with type 2 diabetes (T2DM) in South Korea steadily increased, affecting 87.1% of young adults (19–39 years) and 43.5% of older adults (≥65 years) (1). This rise has been accompanied by an increasing burden of diabetes-related complications, particularly chronic kidney disease (CKD) (2). Epidemiologic studies have reported that obesity is a risk factor for CKD regardless of the presence of T2DM or hypertension (3, 4). Obesity is associated with adverse kidney outcomes, and weight loss can reduce albuminuria and slow eGFR decline (5, 6). Conversely, several studies have reported that underweight status is associated with an increased CKD risk (7, 8), creating controversies on whether excess adiposity or low body weight is more strongly associated with CKD development.

These inconsistent findings may partly reflect age- and sex-related differences in body composition. For instance, women generally have a higher body fat percentage than men, particularly after midlife; men have a greater muscle mass but experience a more pronounced age-related decline in muscle mass (9, 10). Such differences suggest that the relationship between adiposity and CKD risk may vary by age and sex, highlighting the importance of joint age–sex analyses.

Although the body mass index (BMI) is the most widely used measure of obesity in clinical and epidemiological settings, it has considerable limitations because it does not differentiate between fat and lean mass or reflect fat distribution (11). Recently, the Lancet Diabetes & Endocrinology Commission established new diagnostic criteria for clinical obesity by incorporating the clinical confirmation of abnormal body fat mass (12); thus, risk stratification for cardiometabolic outcomes can be performed more accurately (13).

However, large population-based studies on adiposity-related CKD risk in accordance with body-fat–based classifications while jointly considering age and sex differences remain limited. In the present study, the association between adiposity and incident CKD in adults with T2DM was evaluated using the Lancet Commission’s body-fat–based classification; the potential variations in these associations according to age and sex were also examined in a nationwide Korean cohort.

## Research design and methods

### Data source and study population

In this retrospective cohort study, data were collected from the National Health Insurance Service (NHIS) of Korea, which operates a mandatory public insurance program for all citizens and supports public health policies and research activities through the National Health Information Database (NHID) (14). Before the study was initiated, approval was obtained from the Institutional Review Board of Yeungnam University (2025-R-0614). This study was performed in compliance with the Helsinki Declaration of 1975, and the requirement for informed consent was waived.

A total of 2,617,234 Korean individuals who had T2DM (ICD codes E11–E14 with prescribed antidiabetic drugs or fasting blood glucose of ≥126 mg/dL) and underwent at least one general health checkup between January 1, 2015, and December 31, 2016, were enrolled (**Figure 1**). Participants underwent health checkups every two years. The following exclusion criteria were set: 1) participants under 40 years of age (N = 124,179); 2) participants with missing data from health checkups (N = 58,806); 3) participants with eGFR below age-adapted eGFR threshold of CKD determined by age at baseline (15) (N = 105,496); 4) participants with proteinuria of ≥1+ on dipstick urinalysis (N = 142,686); 5) participants with prior end-stage renal disease diagnosis (ICD-10 codes V001, V003, V005, N = 3,319); or 6) participants who had a follow-up health checkup outside the 4 ± 2 years window (N = 358,431). Finally, 1,824,317 participants were included in the final analysis, with a median follow-up of 4 years (Q1–Q3, 3.7–4.3).

**Figure 1:**
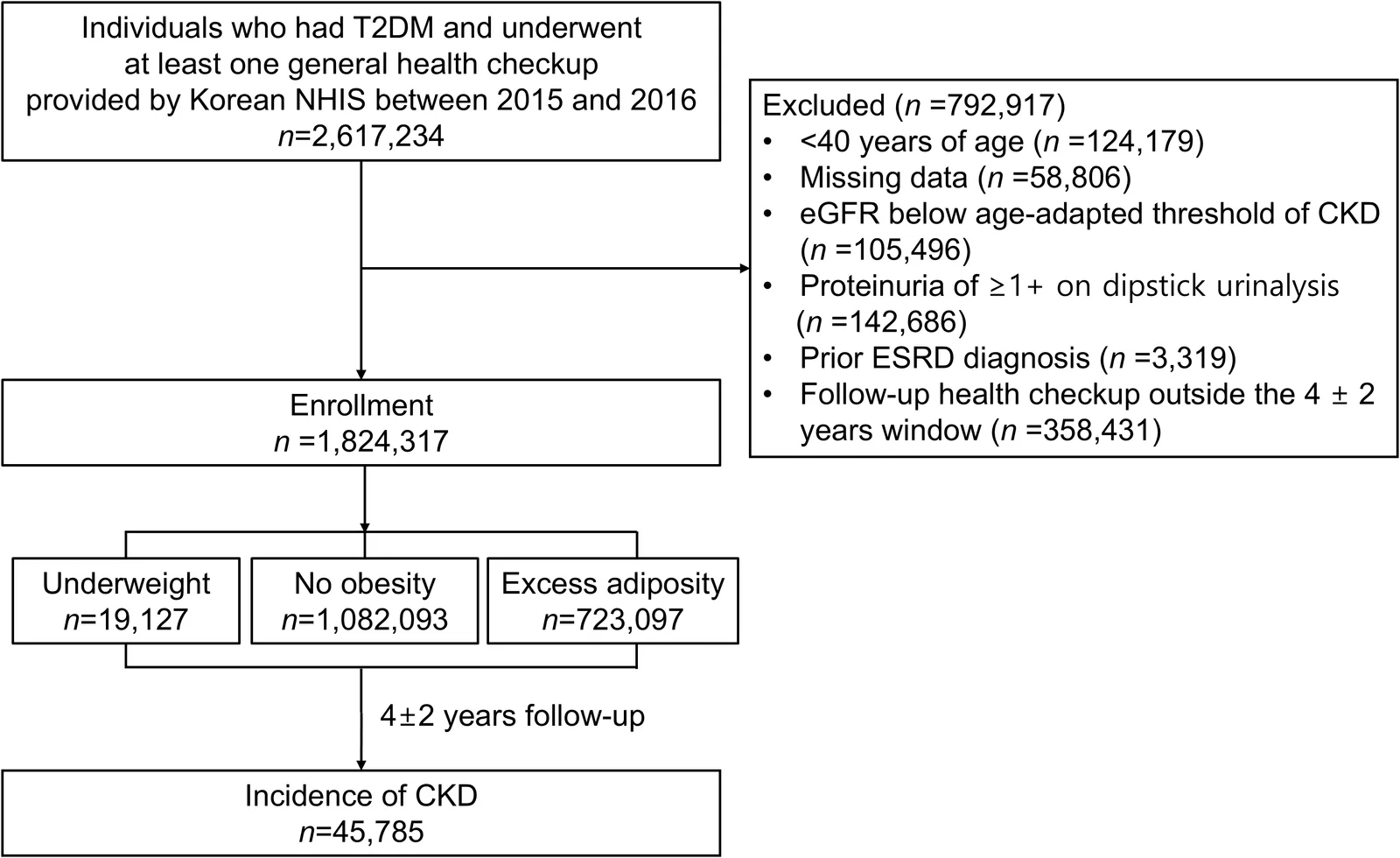
Flowchart of subject inclusion. T2DM, type 2 diabetes mellitus; NHIS, National Health Insurance Service; CKD, chronic kidney disease; ESRD, end-stage renal disease.

### Demographic factors and variables

The following data were collected: age, sex, income, smoking, drinking, exercise status, presence of hypertension, dyslipidemia, use of oral hypoglycemic agents (OHAs), insulin, sodium-glucose-cotransporter-2 inhibitor (SGLT2i), angiotensin-converting-enzyme inhibitor (ACEi), and angiotensin receptor blocker (ARB), diabetes duration, anthropometric measurements, and laboratory parameters at baseline.

Income was based on percentiles and classified into <25th and ≥25th percentiles. Smoking status was categorized as nonsmoker, ex-smoker, or current smoker. Drinking status was classified as non-, mild-, or heavy drinker (alcohol consumption ≥ 30 g/day). Regular exercise was defined as vigorous exercise >3 days/week or moderate-intensity activity >5 days/week. Hypertension was defined in accordance with the ICD-10 code for hypertension (I10–I13, I15), with antihypertensive medications or systolic blood pressure of ≥140 mm Hg and/or diastolic blood pressure of ≥90 mm Hg. Dyslipidemia was defined by the ICD-10 code for dyslipidemia (E78), with lipid-lowering agents or total cholesterol of ≥240 mg/dL. Medication data (OHAs, insulin, SGLT2i, ACEi, and ARB) were based on the prescription status at the baseline health checkup. Diabetes duration was classified as newly diagnosed, <5 years, 5–<10 years, and ≥10 years.

BMI was calculated as weight (kg) divided by height (m^2^). Waist circumference (WC) was measured midway between the lowest rib and the iliac crest. Blood pressure was checked twice in a sitting position, and the mean value was used. Venous blood samples were collected after an overnight fast. Serum glucose, eGFR, total cholesterol, triglycerides, HDL cholesterol, and LDL cholesterol levels were determined.

### Definition of adiposity

Adiposity was defined in accordance with the Lancet Commission’s body-fat–based classification (12). BMI and WC cutoffs were set following the guidelines of the Korean Society for the Study of Obesity (16). The participants with BMI of <18.5 kg/m^2^ without abdominal obesity (WC of <90 cm for Korean men and <85 cm for Korean women) were classified as “underweight”. The participants were classified as “no obesity” if they satisfied one of the following (1): BMI 18.5–25kg/m^2^ without abdominal obesity (2); BMI based obesity (25–40 kg/m^2^ for Korean) without abdominal obesity. “Excess adiposity” served as the foundational category and included all individuals meeting adiposity-related criteria. The participants were considered to have met the criteria if they fulfilled one of the following (1): BMI <40kg/m^2^ with abdominal obesity (WC of ≥90 cm for Korean men and ≥85 cm for Korean women) (2); BMI of ≥40 kg/m^2^ (**Supplementary Table S1**). Data were analyzed using BMI-based classification and compared with the results of a new standard adiposity-based classification.

### Outcome

The primary outcome of this study was incident CKD 4 ± 2 years after the baseline health checkup. Incident CKD was defined using age-adapted eGFR criteria: <60 mL/min/1.73 m^2^ for individuals aged 40–64 years and <45 mL/min/1.73 m^2^ for those aged ≥65 years (15). The threshold was determined by age at follow-up. When the age-adapted threshold was used, the prevalence of CKD in individuals aged ≥70 years was 14.5%; when the uniform CKD definition was used, the prevalence was 33.0% (**Supplementary Table S2**). This approach was used to account for the physiological age-related decline in eGFR, which may otherwise lead to the overestimation of CKD in older adults. eGFR was calculated using the 2021 race-free Chronic Kidney Disease Epidemiology Collaboration equation, which uses standardized serum creatinine, age, and sex without a race coefficient (17). The participants were classified into two groups: incident CKD (CKD^+^) and no CKD (CKD−).

### Statistical analysis

The demographic differences according to incident CKD and adiposity were evaluated using independent t-tests and one way ANOVA for continuous variables and a chi-square test for categorical variables. Because the exact date of CKD onset was unavailable and only baseline and follow-up eGFR measurements were used to ascertain the outcome, time-to-event analyses were not performed. Logistic regression was used to estimate the association between body-composition phenotype and incident CKD identified at follow-up. Odds ratios (ORs) for excess adiposity in relation to CKD were analyzed using logistic regression models. Three models were used: model 1, unadjusted; model 2, adjusted for age, sex, income, smoking, drinking, regular exercise, hypertension, and dyslipidemia; and model 3, adjusted for all variables in model 2 plus the use of three or more OHAs, insulin, diabetes duration, serum glucose levels, and baseline eGFR.

Joint age–sex stratified analyses were performed to examine the potential heterogeneity according to age and sex. Logistic regression models were stratified by age (40–64, ≥65 years) and sex. Additional subgroup analyses were conducted according to the use of SGLT2i, ACEi, and ARB. For each subgroup variable, a global interaction P value was obtained using a likelihood-ratio test comparing models with and without the two interaction terms (2 degrees of freedom). Interactions by age and sex were prespecified, whereas medication-related interaction analyses were exploratory.

All analyses were conducted using SAS version 9.4 (SAS Institute, Cary, NC, USA), and data with p < 0.05 were considered statistically significant. An absolute standardized differences (ASDs) < 0.10 was considered indicative of adequate balance between groups (18).

## Results

### Baseline characteristics

A total of 1,824,317 participants were included in this study. Their mean age was 59.9 ± 10.2 years, and the male-to-female ratio was 1.5:1. The incidence of CKD was 2.5% (n = 45,785). The baseline characteristics between individuals with CKD^+^ and CKD^−^ are presented in **Table 1**. The average age in the CKD^+^ group was significantly higher than those in the CKD^−^ group. The baseline eGFR and the prevalence of heavy drinkers in the CKD^+^ group were lower than those in the CKD^−^ group. The prevalence of hypertension and dyslipidemia and the duration of diabetes in the CKD^+^ group were also higher than those in the CKD^−^ group. The utilization of ≥3 classes of OHAs, insulin, and ARB in the CKD^+^ group was increased compared with that in the CKD^−^ group. The serum glucose levels and utilization of SGLT2i did not differ between the CKD− and CKD+ groups.

**Table 1:** Characteristics of the participants.

	Total	CKD -	CKD +(Incident CKD)	Maximum ASD	Underweight	No obesity	Excess adiposity	Maximum ASD
n	1,824,317	1,778,532	45,785		19,127	1,082,093	723,097	
Baseline eGFR, ml/min/1.73 m^2^	89.9 ± 16.3	90.3 ± 15.9	71.5 ± 17.2	1.16	91.7 ± 16.3	90.4 ± 16.0	88.9 ± 16.2	0.17
Follow-up eGFR, ml/min/1.73 m^2^	87.5 ± 17.4	88.6 ± 16.0	43.8 ± 10.9	3.27	88.8 ± 17.9	88.0 ± 17.0	86.6 ± 17.9	0.12
Age, years	59.9 ± 10.2	59.7 ± 10.2	65.0 ± 10.8	0.51	62.1 ± 11.4	59.6 ± 10.0	60.2 ± 10.5	0.23
40 - 49	3,125,118 (17.1)	3,099,221 (17.4)	25,956 (5.7)	0.37	2,761 (14.4)	180,407 (16.7)	129,350 (17.9)	0.22
50 - 59	5,757,111 (31.6)	5,616,080 (31.6)	141,103 (30.8)		5,460 (28.6)	361,181 (33.4)	209,070 (28.9)	
60 - 69	5,681,124 (31.1)	5,577,090 (31.4)	104,152 (22.8)		5,360 (28.0)	337,798 (31.2)	224,966 (31.1)	
70 - 79	3,220,261 (17.7)	3,080,056 (17.3)	139,730 (30.5)		4,209 (22.0)	177,749 (16.4)	140,068 (19.4)	
≥ 80	459,382 (2.5)	412,372 (2.3)	47,010 (10.3)		1,337 (7.0)	24,958 (2.3)	19,643 (2.7)	
Male, n (%)	1,086,249 (59.5)	1,060,691 (59.6)	25,558 (55.8)	0.08	9,903 (51.8)	662,535 (61.2)	413,811 (57.2)	0.19
Income (<25%), n (%)	382,039 (20.9)	371,812 (20.9)	10,227 (22.3)	0.03	4,827 (25.2)	224,394 (20.7)	152,818 (21.1)	0.11
Smoker, n (%)				0.10				0.25
Non-	1,014,865 (55.6)	987,136 (55.5)	27,729 (60.6)		11,220 (58.7)	593,080 (54.8)	410,565 (56.8)	
Ex-	426,655 (23.4)	416,436 (23.4)	10,129 (22.1)		2,709 (14.2)	251,722 (23.3)	172,134 (23.8)	
Current-	382,887 (21.0)	374,960 (21.1)	7,927 (17.3)		5,198 (27.2)	237,291 (21.9)	140,398 (19.4)	
Drinker, n (%)				0.23				0.21
Non-	1,042,601 (57.2)	1,011,463 (56.9)	31,138 (68.0)		12,763 (66.7)	610,100 (56.4)	419,738 (58.1)	
Mild	612,804 (33.6)	601,195 (33.8)	11,609 (25.4)		4,936 (25.8)	376,390 (34.8)	231,478 (32.0)	
Heavy	168,912 (9.3)	165,874 (9.3)	3,038 (6.6)		1,428 (7.5)	95,603 (8.8)	71,881 (9.9)	
Regular exercise, n (%)	419,073 (22.9)	409,971 (23.1)	9,102 (19.9)	0.08	3,762 (19.7)	269,446 (24.9)	145,865 (20.2)	0.13
HTN, n (%)	1,091,228 (59.8)	1,054,357 (59.3)	36,871 (80.5)	0.48	7,124 (37.3)	575,763 (53.2)	508,341 (70.3)	0.70
Dyslipidemia, n (%)	1,083,062 (59.4)	1,052,025 (59.2)	31,037 (67.8)	0.18	7,337 (38.4)	611,686 (56.5)	463,857 (64.2)	0.53
OHAs ≥3classes, n (%)	379,396 (20.8)	365,357 (20.5)	14,039 (30.7)	0.23	3,881 (20.3)	213,242 (19.7)	162,273 (22.4)	0.07
Insulin use, n (%)	120,848 (6.6)	114,388 (6.4)	6,510 (14.2)	0.26	2,243 (11.7)	69,402 (6.4)	49,203 (6.8)	0.19
SGLT2i, n (%)	32,608 (1.8)	31,857 (1.8)	751 (1.6)	0.01	246 (1.3)	16,270 (1.5)	16,092 (2.2)	0.07
ACEi, n (%)	39,042 (2.1)	37,371 (2.1)	1,671 (3.7)	0.09	393 (2.1)	22,587 (2.1)	10,662 (2.2)	0.01
ARB, n (%)	698,464 (38.3)	671,354 (37.8)	27,110 (59.2)	0.44	3,550 (18.6)	352,009 (32.5)	342,905 (47.4)	0.64
DM duration, n (%)				0.40				0.23
Newly diagnosed	541,483 (29.7)	534,719 (30.1)	6,764 (14.8)		5,583 (29.2)	324,233 (29.9)	211,667 (29.3)	
< 5 years	455,556 (25.0)	446,305 (25.1)	9,251 (20.2)		3,695 (19.3)	257,832 (23.8)	194,029 (26.8)	
5–< 10 years	394,084 (21.6)	383,325 (21.6)	10,759 (23.5)		3,732 (19.5)	230,551 (21.3)	159,801 (22.1)	
≥ 10 years	433,194 (23.7)	414,183 (23.3)	19,011 (41.5)		6,117 (32.0)	269,477 (24.9)	157,600 (21.8)	
BMI, kg/m^2^	25.3 ± 3.4	25.2 ± 3.3	25.3 ± 3.4	0.02	17.5 ± 0.9	23.7 ± 2.2	27.8 ± 3.0	4.68
WC, cm	85.8 ± 8.6	85.8 ± 8.6	86.9 ± 8.8	0.11	69.0 ± 5.8	80.8 ± 5.4	93.8 ± 5.9	4.25
Systolic BP, mmHg	127.9 ± 14.6	127.8 ± 14.5	130.3 ± 15.8	0.17	120.8 ± 15.9	126.5 ± 14.5	130.2 ± 14.4	0.62
Diastolic BP, mmHg	77.9 ± 9.7	77.9 ± 9.7	77.6 ± 10.2	0.02	73.9 ± 10.1	77.1 ± 9.6	79.3 ± 9.7	0.54
Glucose, mg/dL	142.3 ± 42.1	142.3 ± 41.8	141.9 ± 51.4	0.01	146.8 ± 59.1	142.4 ± 42.8	142.1 ± 40.5	0.09
Total cholesterol, mg/dL	184.6 ± 42.9	184.8 ± 42.9	177.8 ± 43.6	0.16	177.9 ± 39.8	184.5 ± 42.8	184.9 ± 43.1	0.17
HDL cholesterol, mg/dL	51.3 ± 14.6	51.3 ± 14.6	48.6 ± 14.0	0.19	60.3 ± 18.3	52.2 ± 15.1	49.6 ± 13.3	0.66
LDL cholesterol, mg/dL	102.9 ± 38.0	103.1 ± 37.9	96.2 ± 37.8	0.18	96.3 ± 35.1	103.3 ± 37.8	102.4 ± 38.3	0.19
Triglyceride, mg/dL	134.5 (116.7–154.7)	134.2 (116.8–134.8)	146.7 (131.9–147.4)	1.46	92.0 (91.3–92.7)	127.2 (127.1–127.4)	147.7 (147.5–149.8)	0.89

Chronic kidney disease (CKD) is defined as eGFR <60 mL/min/1.73 m^2^ (age 40-64) and eGFR <45 mL/min/1.73 m^2^ (age ≥65), measured four years after the baseline health checkup.

Underweight, BMI<18.5kg/m^2^ without abdominal obesity (WC <90 cm for men and <85 cm for women); No obesity, (1) BMI 18.5–25 kg/m^2^ without abdominal obesity OR (2) BMI 25–40kg/m^2^ without abdominal obesity; Excess adiposity, (1) BMI <40 kg/m^2^ with abdominal obesity (WC ≥90 cm for men and ≥85 cm for women) OR (2) BMI ≥40kg/m^2^.

eGFR, estimated glomerular filtration rate; BMI, body mass index; WC, waist circumference; M, male; W, women; HTN, hypertension; OHA, oral hypoglycemic agents; DM, diabetes mellitus; BP, blood pressure; HDL, high-density lipoprotein; LDL, low-density lipoprotein.

Medication data were based on the prescription status between the baseline and follow-up health checkup.

Continuous variables are presented as means ± SD, and categorical variables are presented as percentages. TG values are presented as the geometric mean (95% CI).

### CKD risk according to adiposity

The participants were categorized as underweight (1.0%, n=19,127), no obesity (59.3%, n=1,082,093), and excess adiposity (39.6%, n=723,097). The individuals in the underweight group were older and had higher rates of insulin use, and longer diabetes duration. The individuals with excess adiposity had a higher prevalence of hypertension and dyslipidemia and greater use of ARBs. The serum glucose levels and utilization of ≥3 OHAs, SGLT2i, and ACEi did not differ among groups (**Table 1**).

The incidence and risk of CKD according to adiposity are presented in **Table 2**. The highest CKD incidence was observed in the excess adiposity group (2.9%), followed by the underweight (2.8%) and no obesity (2.3%) groups. After full adjustment, both underweight and excess adiposity were associated with an increased risk of CKD compared with the no obesity group; however, the association was substantially stronger for underweight (OR 1.41, 95% CI 1.29–1.54) than for excess adiposity (OR 1.03, 95% CI 1.01–1.05). After excluding participants with conditions potentially associated with underweight—malignant neoplasms (ICD-10 codes C00–C97), chronic obstructive pulmonary disease (J43.x or J44), alcoholic liver disease (K70), liver fibrosis or cirrhosis (K74), cachexia (R64), and muscle wasting or atrophy (M62.5x)—the association between underweight and increased CKD risk remained significant (OR 1.42, 95% CI 1.24–1.62), supporting the robustness of our primary findings.

**Table 2:** Incidence and risk of CKD (using age-adapted threshold of eGFR) according to adiposity-based classification.

Adiposity	Total, n	CKD, n (%)	Model 1 OR (95% CI)	Model 2 OR (95% CI)	Model 3 OR (95% CI)
Underweight	19,127	531 (2.8)	1.23 (1.13,1.34)	1.22 (1.12,1.33)	1.41 (1.29,1.54)
No Obesity	1,082,093	24,491 (2.3)	1 (Ref.)	1 (Ref.)	1 (Ref.)
Excess Adiposity	723,097	20,763 (2.9)	1.287 (1.25,1.30)	1.10 (1.08,1.12)	1.03 (1.01,1.05)
p-value			<0.001	<0.001	<0.001
After exclusion of major-wasting associated conditions*					
Underweight	10,116	247 (2.4)	1.22 (1.08,1.39)	1.27 (1.12,1.45)	1.42 (1.24,1.62)
No Obesity	666,768	13,359 (2.0)	1 (Ref.)	1 (Ref.)	1 (Ref.)
Excess Adiposity	421,345	10,635 (2.5)	1.27 (1.23,1.3)	1.09 (1.06,1.12)	1.02 (1.00,1.05)
p-value			<0.001	<0.001	<0.001

Chronic kidney disease (CKD) is defined as eGFR <60 mL/min/1.73 m^2^ (age 40-64) and eGFR <45 mL/min/1.73 m^2^ (age ≥65), measured 4 years after the baseline health checkup.

Underweight, BMI<18.5kg/m^2^ without abdominal obesity (WC <90 cm for men and <85 cm for women); No obesity, (1) BMI 18.5–25 kg/m^2^ without abdominal obesity OR (2) BMI 25–40kg/m^2^ without abdominal obesity; Excess adiposity, (1) BMI <40 kg/m^2^ with abdominal obesity (WC ≥90 cm for men and ≥85 cm for women) OR (2) BMI ≥40kg/m^2^.

Logistic regression analysis was performed. Model 1, unadjusted; Model 2, adjusted for age, sex, income, smoking, drinking, regular exercise, hypertension, and dyslipidemia; Model 3, adjusted for all variables in model 2 plus the use of three or more OHAs, insulin, diabetes duration, serum glucose levels, and baseline eGFR.

*Participants with major wasting-associated conditions were excluded, including malignant neoplasms (ICD-10 codes C00–C97), chronic obstructive pulmonary disease (J43.x or J44), alcoholic liver disease (K70), liver fibrosis or cirrhosis (K74), cachexia (R64), and muscle wasting or atrophy (M62.5x).

Across sensitivity analyses using repeated age-adapted threshold of CKD development and a ≥40% decline in eGFR (19), underweight was consistently associated with an increased risk of incident CKD or clinically meaningful kidney function decline (**Supplementary Table S3**).

When BMI-based classification was used instead of adiposity-based classification, underweight remained associated with the increased CKD risk, whereas obesity was inversely related to the CKD risk (**Supplementary Table S4**). When a uniform eGFR threshold was applied instead of age-adapted criteria, the increased CKD risk was associated with excess adiposity but not with underweight (**Supplementary Table S5**) possibly because the higher prevalence of CKD under the uniform definition might obscure the identification of risk factors.

### Subgroup analysis

The risk of adiposity on incident CKD according to age (40–64, ≥65 years), sex (male and female), and utilization of renoprotective medications (SGLT2i, ACEi, and ARB) was analyzed (**Table 3**). Underweight was significantly associated with an increased risk of incident CKD in both age groups compared with no obesity, with a more pronounced association among individuals aged 40–64 years. Similarly, underweight was associated with an increased risk of incident CKD in both men and women. In contrast, excess adiposity was associated with an increased risk of incident CKD among women, whereas this association was not observed among men. In analyses stratified by the use of renoprotective medications, underweight remained significantly associated with an increased risk of incident CKD regardless of medication use. Joint age–sex stratified analyses were performed to further clarify this heterogeneity. With BMI-only classification, obesity was slightly inversely associated with CKD in men, whereas a modest increase in CKD risk was observed in women aged ≥65 years (**Supplementary Figure S1**). In contrast, under the classification incorporating both BMI and WC (Lancet Commission classification), excess adiposity was significantly associated with CKD in women in both age groups (**Figure 2**), suggesting that incorporating WC may better capture adiposity-related CKD risk than BMI alone. Underweight showed the strongest positive association with CKD under both classifications, particularly among men aged 40–64 years.

**Table 3:** Adjusted associations and interactions of adiposity with incident CKD by age, sex, and use of SGLT2i, ACEi, and ARB.

Subgroup		N	CKD (%)	OR (95% CI)	P for interaction	Adjusted risk (%)	Adjusted risk difference
Age, 40 - 64	Underweight	11,370	241 (2.1)	1.57 (1.37–1.79)	<0.001	2.65	0.94
	No obesity	748,212	12,760 (1.7)	1.00 (Ref.)		1.71	Ref.
	Excess adiposity	470,488	9,386 (2.0)	1.00 (0.97–1.03)		1.71	0.01
Age, ≥ 65	Underweight	7,757	290 (3.7)	1.28 (1.13–1.46)		4.47	0.96
	No obesity	333,845	11,731 (3.5)	1.00 (Ref.)		3.51	Ref.
	Excess adiposity	252,616	11,377 (4.5)	1.07 (1.04–1.10)		3.76	0.25
Sex, Male	Underweight	9,903	268 (2.7)	1.44 (1.27–1.64)	<0.001	3.14	0.94
	No obesity	662,535	14,597 (2.2)	1.00 (Ref.)		2.20	Ref.
	Excess adiposity	413,811	10,693 (2.6)	0.97 (0.95–1.00)		2.15	-0.06
Sex, Female	Underweight	9,224	263 (2.9)	1.40 (1.23–1.59)		3.26	0.91
	No obesity	419,558	9,894 (2.4)	1.00 (Ref.)		2.36	Ref.
	Excess adiposity	309,286	10,070 (3.3)	1.10 (1.07–1.14)		2.59	0.23
SGLTi, No	Underweight	18,881	519 (2.8)	1.39 (1.27–1.53)	0.008	3.13	0.86
	No obesity	1,065,823	24,122 (2.3)	1.00 (Ref.)		2.26	Ref.
	Excess adiposity	707,005	20,393 (2.9)	1.03 (1.01–1.05)		2.33	0.07
SGLTi, Yes	Underweight	246	12 (4.9)	2.90 (1.54–5.46)		6.31	4.05
	No obesity	16,270	369 (2.3)	1.00 (Ref.)		2.27	Ref.
	Excess adiposity	1,601	370 (2.3)	0.90 (0.77–1.04)		2.04	-0.23
ACEi, No	Underweight	18,734	509 (2.7)	1.42 (1.29–1.56)	0.666	3.13	0.90
	No obesity	1,059,056	23,575 (2.2)	1.00 (Ref.)		2.23	Ref.
	Excess adiposity	707,035	20,030 (2.8)	1.03 (1.01–1.05)		2.28	0.06
ACEi, Yes	Underweight	393	22 (5.6)	1.22 (0.77–1.94)		4.92	0.86
	No obesity	22,587	916 (4.1)	1.00 (Ref.)		4.06	Ref.
	Excess adiposity	16,062	333 (2.1)	1.07 (0.96–1.18)		4.29	0.23
ARB, No	Underweight	15,577	333 (2.1)	1.45 (1.29–1.62)	0.683	2.24	0.68
	No obesity	730,084	11,388 (1.6)	1.00 (Ref.)		1.56	Ref.
	Excess adiposity	380,192	6,954 (1.8)	1.01 (0.98–1.04)		1.58	0.02
ARB, Yes	Underweight	3,550	198 (5.6)	1.38 (1.18–1.60)		5.05	1.33
	No obesity	352,009	13,103 (3.7)	1.00 (Ref.)		3.72	Ref.
	Excess adiposity	342,905	13,809 (4.0)	1.02 (1.00–1.05)		3.81	0.09

Chronic kidney disease (CKD) is defined as eGFR <60 mL/min/1.73 m^2^ (age 40-64) and eGFR <45 mL/min/1.73 m^2^ (age ≥65), measured 4 years after the baseline health checkup.

Underweight, BMI<18.5kg/m^2^ without abdominal obesity (WC <90 }cm for men and <85 cm for women); No obesity, (1) BMI 18.5–25 kg/m^2^ without abdominal obesity OR (2) BMI 25–40kg/m^2^ without abdominal obesity; Excess adiposity, (1) BMI <40 kg/m^2^ with abdominal obesity (WC ≥90 cm for men and ≥85 cm for women) OR (2) BMI ≥40kg/m^2^.

Logistic regression analysis was performed. Each model was adjusted for age, sex, income, smoking, drinking, regular exercise, hypertension, dyslipidemia, use of three or more OHAs, insulin, diabetes duration, serum glucose levels, and baseline eGFR.

P values for interaction are global P values derived from likelihood-ratio tests of the interaction between adiposity category and each subgroup variable (2 degrees of freedom).

**Figure 2:**
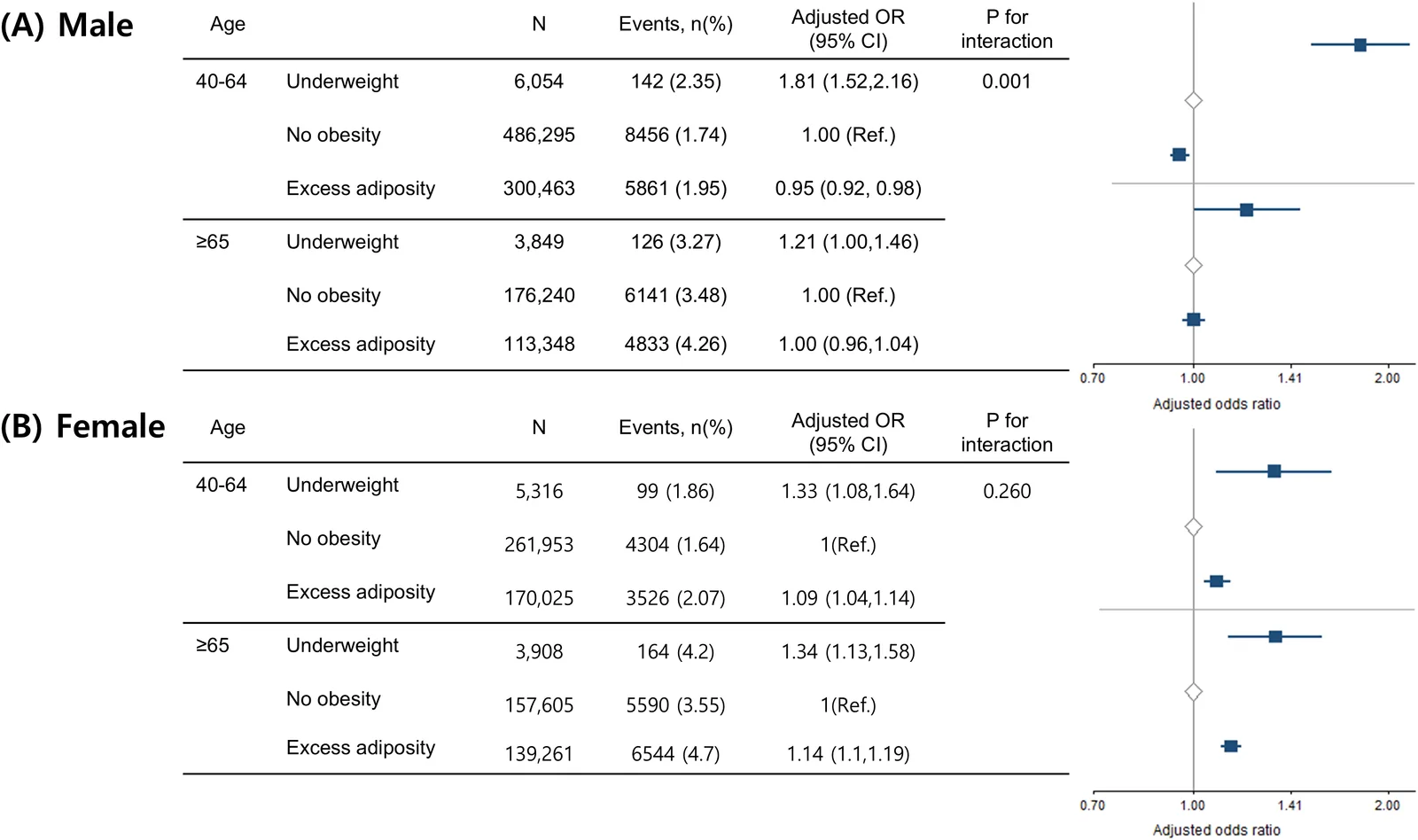
Incidence of CKD according to age and adiposity among **(A)** males and **(B)** females. Logistic regression analysis was performed. Squares indicate adjusted odds ratios, and horizontal lines indicate 95% confidence intervals. Chronic kidney disease (CKD) is defined as eGFR <60 mL/min/1.73 m^2^ (age 40-64) and eGFR <45 mL/min/1.73 m^2^ (age ≥65), measured 4 years after the baseline health checkup. Underweight, BMI<18.5kg/m^2^ without abdominal obesity (WC <90 cm for men and <85 cm for women); No obesity, (1) BMI 18.5–25 kg/m^2^ without abdominal obesity OR (2) BMI 25–40kg/m^2^ without abdominal obesity; Excess adiposity, (1) BMI <40 kg/m^2^ with abdominal obesity (WC ≥90 cm for men and ≥85 cm for women) OR (2) BMI ≥40kg/m^2^. Each model was adjusted for age, sex, income, smoking, drinking, regular exercise, hypertension, dyslipidemia, use of three or more OHAs, insulin, diabetes duration, serum glucose levels, and baseline eGFR.

## Discussion

This large-scale, 4-year study of over 1.8 million Korean adults aged ≥40 years with T2DM examined the association of adiposity with incident CKD using the Lancet Commission body-fat–based classification and age-adapted eGFR criteria. In fully adjusted models, underweight was consistently associated with an increased risk of CKD. Conversely, excess adiposity showed a modest overall association with increased CKD risk but substantial age- and sex-specific heterogeneity. An increased risk was observed predominantly in women, whereas an inverse association was observed in middle-aged men. Importantly, incorporating WC with BMI appeared to better capture adiposity-related CKD risk, particularly among women, than BMI-based classification alone. These findings highlight the importance of considering adiposity beyond BMI alone, together with age- and sex-specific differences, when assessing CKD risk in individuals with T2DM.Several clinical studies have investigated the association between BMI-based obesity and CKD but have not accounted for excess adiposity (20, 21). In our study, excess adiposity was observed in 39.6% of the population, whereas BMI-defined obesity was present in 50.6% (**Supplementary Table S4**). These results indicated that the discordance between BMI- and adiposity-based classification was substantial. They also highlighted the potential for misclassification when obesity was defined solely by BMI. Given that body composition, including reduced muscle mass and increased visceral fat, may influence the CKD risk independently of BMI (6), incorporating adiposity-based classification may provide more accurate CKD risk stratification than conventional BMI categories. In addition to WC, alternative anthropometric indices, including waist-to-height ratio, A Body Shape Index, Body Roundness Index, and Weight-Adjusted Waist Index, may provide complementary information on central adiposity, body shape, and abdominal adiposity independent of overall body weight (22–25).

In the present study, which applied the Lancet Commission classification, underweight emerged as an independent high-risk phenotype for incident CKD in all participants. Evidence from two large nationwide Korean NHIS cohort studies has consistently demonstrated that underweight (BMI < 18.5 kg/m^2^) significantly increases the risk of progression to end-stage kidney disease in the general population (7) and more prominently among individuals with type 2 diabetes (8). The association between underweight status and incident CKD may reflect several non-mutually exclusive pathways. Being underweight may reflect a metabolically and structurally vulnerable state that predisposes to accelerated renal function decline potentially via protein–energy wasting, sarcopenia, and chronic inflammation (7, 26, 27). Serum albumin has antioxidant and anti-inflammatory properties and may also reflect nutritional status, systemic inflammation, and overall illness severity (28, 29). However, direct measures of skeletal muscle mass, visceral and subcutaneous adipose tissue, serum albumin, and inflammatory biomarkers were unavailable in the present study, and this hypothesis could not be directly evaluated. Accordingly, in CKD risk stratification and prevention among patients with type 2 diabetes, careful assessment of underweight and weight loss should be prioritized alongside the traditional focus on obesity. In contrast to the consistent risk observed with underweight, the association between excess adiposity and CKD varied substantially by age and sex in joint age- and sex-stratified analyses, indicating that the renal impact of excess adiposity may be context dependent rather than uniform. This pattern partly reflected sex-specific changes in body composition during midlife. Given that females generally have a higher fat proportion than males at comparable BMI levels (30, 31), particularly after midlife, the metabolic burden associated with excess adiposity may be relatively greater, contributing to their consistently increased CKD risk. In middle-aged males, the inverse association may reflect biological and methodological factors. At comparable BMI levels, males typically have greater lean mass and a lower fat proportion than females; this characteristic may partially buffer adiposity-related metabolic stress (32, 33). During a relatively limited follow-up period, obesity-associated glomerular hyperfiltration in earlier stages of metabolic disease may transiently preserve eGFR above diagnostic thresholds (34). Notably, in a sensitivity analysis using a ≥40% decline in eGFR from baseline as the outcome, excess adiposity was associated with an increased risk of kidney function decline, suggesting that hyperfiltration-related preservation of eGFR may not fully explain the observed association (**Supplementary Table S3**).

In subgroup analyses by SGLT2i, ACEi, and ARB use, underweight remained associated with higher incident CKD risk regardless of medication use. These results should be interpreted cautiously because treatment was not randomly assigned and may reflect differences in clinical characteristics, indications, baseline kidney risk, or unmeasured factors. Because only 0.03% of participants used glucagon-like peptide-1 receptor agonists (data not shown), associations by GLP-1 receptor agonist use could not be meaningfully assessed. Future studies using contemporary databases should distinguish treatment-related intentional weight loss from low body weight related to frailty, sarcopenia, poor nutritional reserve, or unintentional weight loss when evaluating kidney outcomes.

This study has several limitations. First, CKD was defined on the basis of eGFR without incorporating albuminuria measurements. Because albuminuria is an important marker of kidney damage, early or specific CKD phenotypes may be misclassified without this information. Second, direct body fat distribution measurements were unavailable. Although WC is a validated surrogate for abdominal adiposity, more precise imaging-based assessments of visceral and subcutaneous fat can provide additional mechanistic insights. Third, potential selection bias may have arisen because the analysis was restricted to participants who underwent a repeat national health examination within the prespecified follow-up window. Although participation in biennial national health checkups is widespread among Korean adults aged ≥40 years, repeat screening attendance may still be associated with health status, healthcare utilization, survival, and socioeconomic factors; therefore, selection bias cannot be completely excluded. Fourth, although individuals with type 1 diabetes mellitus (ICD code E10) were excluded, our claims- and laboratory-based selection algorithm may not have completely excluded latent autoimmune diabetes in adults, or secondary diabetes due to endocrine disorders. Fifth, the study population consisted exclusively of Korean individuals, possibly limiting generalizability to other ethnic groups with different body composition patterns and CKD risk profiles. Lastly, this observational study cannot establish causality, so the associations between adiposity categories and incident CKD should be interpreted as associations rather than proof of direct renal effects. The proposed mechanisms should consequently be regarded as biologically plausible hypotheses rather than mechanisms established by the present data.

Despite these limitations, this study has several notable strengths. First, a nationally representative, high-quality health database from Korea was used; as such, the robustness and external validity of the findings within this population were enhanced. Second, incident CKD could be reliably assessed over time because of the approximately 4-year follow-up period. Importantly, this study demonstrated that the association between adiposity and CKD differed according to age and sex in patients with T2DM, highlighting the heterogeneous renal impact of adiposity and the need for individualized risk stratification. Third, the Lancet Commission classification framework was incorporated to more accurately categorize excess adiposity beyond BMI alone. Finally, CKD was defined using age-adapted eGFR thresholds to reduce the potential overestimation of CKD among older adults. With this approach, CKD prevalence in individuals aged ≥70 years decreased from 33.0% (with a uniform definition) to 14.5% (**Supplementary Table S2**). Therefore, age-specific kidney function thresholds are relevant in epidemiological studies.

In conclusion, the underweight status of individuals with T2DM was consistently associated with an increased risk of incident CKD in this nationwide cohort. Conversely, the association between excess adiposity and CKD showed marked heterogeneity by age and sex. The risk was increased in females, whereas an inverse association was observed in middle-aged males. Given that body composition changes with age and sex, assessing the CKD risk in T2DM should include joint age–sex evaluation and adiposity-based measures beyond BMI, thus highlighting the importance of personalized CKD risk stratification and targeted renoprotective strategies.

## Data Availability

The data analyzed in this study is subject to the following licenses/restrictions: Raw data are available to researchers upon relevant request, with approval by the Korean National Health Insurance Sharing Service. Requests to access these datasets should be directed to https://nhiss.nhis.or.kr/ (accession number: NHIS-2025-12-1-030).
