# Supplementary material for: Age- and sex-specific associations of underweight and excess adiposity with incident CKD in type 2 diabetes: nationwide Korean cohort study
Source: Front Endocrinol (Lausanne). 2026 Sep 17;17:1928884. doi: 10.3389/fendo.2026.1928884 (PMC13626875; doi:10.3389/fendo.2026.1928884)
Supplement: Supplementary file 1 [file SupplementaryFile1.docx]

**Supplementary Table 1. Cross-classification of body mass index and waist circumference used to define adiposity categories**

| **BMI categories** | **Normal Waist Circumference**  **(<90 cm for men and <85 cm for women)** | **Abdominal obesity**  **(≥90 cm for men and ≥85 cm for women)** |
| --- | --- | --- |
| **<18.5 kg/m^2^** | Underweight  (n=19,127) | Excess adiposity  (n=156) |
| **18.5–25kg/m^2^** | No obesity  (n=777,399) | Excess adiposity  (n=104,217) |
| **25–40 kg/m^2^** | No obesity  (n=304,694) | Excess adiposity  (n=616,670) |
| **≥40 kg/m^2^** | Excess adiposity  (n=46) | Excess adiposity  (n=2,008) |

**Supplementary Table 2. Prevalence of CKD according to eGFR threshold (age-adapted vs. uniform definition)**

|  | **Age-adapted threshold** | | **Uniform definition** | |
| --- | --- | --- | --- | --- |
| Age | Total | CKD | Total | CKD |
| **40 - 49** | 312,518 | 2,596(0.8%) | 312,501 | 2,596(0.8%) |
| **50 - 59** | 575,711 | 14,103(2.4%) | 575,678 | 14,114(2.5%) |
| **60 - 69** | 568,124 | 10,415(1.8%) | 554,872 | 35,171(6.3%) |
| **70 - 79** | 322,026 | 13,970(4.3%) | 278,181 | 39,373(14.2%) |
| **≥ 80** | 45,938 | 4,701(10.2%) | 35,400 | 8,626(18.8%) |
| **Overall** | 1,824,317 | 45,785(2.5%) | 1,656,752 | 99,880(6.0%) |

Age-adapted threshold: eGFR <60 mL/min/1.73 m^2^ (age 40-64) and eGFR <45 mL/min/1.73 m^2^ (age ≥65)

Uniform definition: eGFR <60 mL/min/1.73 m^2^ across all age groups

**Supplementary Table 3. Incidence and risk of CKD and kidney function decline according to adiposity-based classification**

|  | **CKD (using repeated age-adapted threshold of CKD development)** | | | **Kidney function decline (≥40% reduction in eGFR)** | | |
| --- | --- | --- | --- | --- | --- | --- |
|  | **Total, n** | **Event, n(%)** | **Adjusted OR (95% CI)** | **Total, n** | **Event, n(%)** | **Adjusted OR (95% CI)** |
| **Underweight** | 13,894 | 155(1.12) | 1.56(1.32,1.85) | 19,127 | 435(2.3) | 1.12(1.01,1.23) |
| **No Obesity** | 932,050 | 8,245(0.88) | 1(Ref.) | 1,082,093 | 17633(1.6) | 1(Ref.) |
| **Excess Adiposity** | 609,445 | 7,009(1.15) | 1.03(0.99,1.06) | 723,097 | 13979(1.9) | 1.11(1.08,1.13) |
| **p-value** |  |  | <0.001 |  |  | <0.001 |

Chronic kidney disease (CKD) is defined as eGFR <60 mL/min/1.73 m^2^ (age 40-64) and eGFR <45 mL/min/1.73 m^2^ (age ≥65), at two or more follow-up health checkups. Kidney function decline was defined as a ≥40% reduction in eGFR from baseline.

Underweight, BMI<18.5kg/m^2^ without abdominal obesity (WC <90 cm for men and <85 cm for women); No obesity, (1) BMI 18.5–25 kg/m^2^ without abdominal obesity OR (2) BMI 25–40kg/m^2^ without abdominal obesity; Excess adiposity, (1) BMI <40 kg/m^2^ with abdominal obesity (WC ≥90 cm for men and ≥85 cm for women) OR (2) BMI ≥40kg/m^2^.

Logistic regression analysis was performed, after adjusted for age, sex, income, smoking, drinking, regular exercise, hypertension, and dyslipidemia, the use of three or more OHAs, insulin, diabetes duration, serum glucose levels, and baseline eGFR

**Supplementary Table 4. Incidence and risk of CKD (using age-adapted threshold of eGFR) according to BMI-based classification**

|  | **Total, n** | **CKD, n(%)** | **Model 1**  **OR (95% CI)** | **Model 2**  **OR (95% CI)** | **Model 3**  **OR (95% CI)** |
| --- | --- | --- | --- | --- | --- |
| **Underweight** | 19,283 | 541(2.81) | 1.15(1.05,1.25) | 1.20(1.10,1.31) | 1.39(1.27,1.52) |
| **No Obesity** | 881,616 | 21630(2.45) | 1(Ref.) | 1(Ref.) | 1(Ref.) |
| **Obesity** | 923,418 | 23614(2.56) | 1.04(1.02,1.06) | 1.04(1.02,1.06) | 0.98(0.96,0.998) |
| **p-value** |  |  | <0.001 | <0.001 | <0.001 |

Chronic kidney disease (CKD) is defined as eGFR <60 mL/min/1.73 m^2^ (age 40-64) and eGFR <45 mL/min/1.73 m^2^ (age ≥65), measured 4 years after the baseline health checkup.

Underweight, BMI<18.5kg/m^2^; No obesity, BMI 18.5~25kg/m^2^; Obesity, BMI≥25kg/m^2^

Logistic regression analysis was performed. Model 1, unadjusted; Model 2, adjusted for age, sex, income, smoking, drinking, regular exercise, hypertension, and dyslipidemia; Model 3, adjusted for all variables in model 2 plus the use of three or more OHAs, insulin, diabetes duration, serum glucose levels, and baseline eGFR

**Supplementary Table 5. Incidence and risk of CKD (using uniform threshold of eGFR) according to adiposity-based classification**

|  | **Total, n** | **CKD, n(%)** | **Model 1**  **OR (95% CI)** | **Model 2**  **OR (95% CI)** | **Model 3**  **OR (95% CI)** |
| --- | --- | --- | --- | --- | --- |
| **Underweight** | 18,455 | 1,062(5.75) | 1.13(1.06,1.20) | 0.91(0.85,0.97) | 1.04(0.97,1.11) |
| **No Obesity** | 1,048,045 | 53,785(5.13) | 1(Ref.) | 1(Ref.) | 1(Ref.) |
| **Excess Adiposity** | 690,132 | 45,033(6.53) | 1.29(1.27,1.31) | 1.12(1.11,1.14) | 1.09(1.08,1.11) |
| **p-value** |  |  | <0.001 | <0.001 | <0.001 |

Chronic kidney disease (CKD) is defined as eGFR <60 mL/min/1.73 m^2^, measured 4 years after the baseline health checkup.

Underweight, BMI<18.5kg/m^2^ without abdominal obesity (WC <90 cm for men and <85 cm for women); No obesity, (1) BMI 18.5–25 kg/m^2^ without abdominal obesity OR (2) BMI 25–40kg/m^2^ without abdominal obesity; Excess adiposity, (1) BMI <40 kg/m^2^ with abdominal obesity (WC ≥90 cm for men and ≥85 cm for women) OR (2) BMI ≥40kg/m^2^.

Logistic regression analysis was performed. Model 1, unadjusted; Model 2, adjusted for age, sex, income, smoking, drinking, regular exercise, hypertension, and dyslipidemia; Model 3, adjusted for all variables in model 2 plus the use of three or more OHAs, insulin, diabetes duration, serum glucose levels, and baseline eGFR

**Supplementary Figure 1. Incidence of CKD according to age and BMI-defined obesity among (A)male and (B)female**

Logistic regression analysis was performed. Squares indicate adjusted odds ratios, and horizontal lines indicate 95% confidence intervals.

Chronic kidney disease (CKD) is defined as eGFR <60 mL/min/1.73 m^2^ (age 40-64) and eGFR <45 mL/min/1.73 m^2^ (age ≥65), measured 4 years after the baseline health checkup.

Underweight, BMI<18.5kg/m^2^; No obesity, BMI 18.5~25kg/m^2^; Obesity, BMI≥25kg/m^2^

Each model was adjusted for age, sex, income, smoking, drinking, regular exercise, hypertension, dyslipidemia, use of three or more OHAs, insulin, diabetes duration, serum glucose levels, and baseline eGFR.
